# Association of DNA repair gene variants with colorectal cancer: risk, toxicity, and survival

**DOI:** 10.1186/s12885-020-06924-z

**Published:** 2020-05-12

**Authors:** Hamideh Salimzadeh, Elinor Bexe Lindskog, Bengt Gustavsson, Yvonne Wettergren, David Ljungman

**Affiliations:** 1 Department of Surgery, Institute of Clinical Sciences, Sahlgrenska Academy, University of Gothenburg, Sahlgrenska University Hospital, Östra, 416 85 Gothenburg, Sweden; 2 Digestive Oncology Research Centre, Digestive Disease Research Institute, Tehran University of Medical Sciences, Tehran, Iran; 3 Region Västra Götaland, Department of Surgery, Sahlgrenska University Hospital, Gothenburg, Sweden

**Keywords:** XRCC1, ERCC1, ERCC2, Colorectal cancer, Toxicity

## Abstract

**Background:**

Single nucleotide polymorphisms (SNPs) in DNA repair genes have a potential clinical value in predicting treatment outcomes. In the current study, we examined the association of SNPs in the genes XRCC1-rs25487, ERCC1-rs11615, ERCC2-rs238406, and ERCC2-rs13181 with colorectal cancer (CRC) risk, relapse-free survival (RFS), overall survival (OS), and toxicity during chemotherapy.

**Methods:**

SNPs were analysed in 590 CRC cases and 300 controls using TaqMan technology. The association of SNPs with CRC risk and toxicity during chemotherapy was analysed using Chi2 test. The Kaplan–Meier method and log-rank test was used to measure the effects of the SNPs on RFS and OS.

**Results:**

The CC genotype of ERCC2-rs238406 and the ERCC2-rs13181 C allele were associated with a significantly increased risk of CRC. The ERCC1-rs11615 genotype T/T was associated with stomatitis in adjuvant chemotherapy (*p* = 0.03). Also, more patients with the ERCC2-rs13181 C allele needed dose reduction compared to patients with the A/A genotype (*p* = 0.02). In first line chemotherapy, more patients with the ERCC1-rs11615 C allele suffered from nausea compared to those with the T/T genotype (*p* = 0.04) and eye reactions and thrombocytopenia were more common in patients with the ERCC2-rs13181 C allele compared to the A/A genotype (*p* = 0.006 and *p* = 0.004, respectively). ERCC2- rs238406 C/C was also associated with a higher frequency of thrombocytopenia (*p* = 0.03). A shorter 5-year OS was detected in stage I & II CRC patients with the ERCC2- rs238406 C allele (*p* = 0.02). However, there was no significant association between the SNPs and 5-year RFS.

**Conclusions:**

Both SNPs in ERCC2 were associated with risk of CRC as well as toxicity during first line treatment. In addition, ERCC2- rs238406 was linked to OS in early stage CRC. The ERCC1-rs11615 variant was associated with toxicity during adjuvant chemotherapy. The results add support to previous findings that SNPs in ERCC1 and ERCC2 have a prognostic and predictive value in clinical management of CRC.

## Background

Colorectal cancer (CRC) is a major health concern with approximately 1.8 million incident cases each year worldwide [1]. Although survival of CRC has been improved with novel chemotherapeutic drugs [2], chemotherapy has not increased the overall survival (OS) in advanced CRC dramatically [3]. A meta-analysis confirmed a significantly improved tumor response rate (23% vs 11%) in FLV therapy; that is, 5-fluorouracil (5-FU) with leucovorin versus 5-FU alone [4]. Moreover, adding oxaliplatin to FLV significantly improved OS in the adjuvant treatment of stage II/III CRC patients [5] and is currently considered the standard therapy for first-line treatment of metastatic CRC, with a response rate of over 40% [6]. However, oxaliplatin-based treatment is hampered by the serious drawback of tumor cell drug resistance, in which DNA- repair plays a key role [7].

A number of single nucleotide polymorphisms (SNPs) in DNA- repair genes are known to affect cancer susceptibility, prognosis, and therapeutic outcomes [8]. Indeed, SNPs in drug-targeted genes [9], metabolizing enzymes [10], and DNA-repair enzymes [11] have been linked to inter-individual differences in the efficacy and toxicity of numerous drugs. Excision repair cross-complementing group 1 (ERCC1) and 2 (ERCC2) and X-ray repair cross-complementing group 1 (XRCC1) are DNA repair enzymes which play important roles in nucleotide excision repair [8].

The ERCC1 and ERCC2 proteins are highly conserved enzymes [12] which participate in the key steps of nucleotide excision repair such as the damage recognition and removal of DNA lesions induced by substances such as platinum [8, 13, 14]. SNPs in the ERCC1 and ERCC2 genes might be useful as predictive factors for oxaliplatin-based chemotherapy [15]. For instance, the common ERCC1 rs11615 variant, which results in the synonymous variant Asn118Asn, is associated with increased mRNA and protein levels affecting repair of oxaliplatin-induced DNA lesions [16]. In the ERCC2 gene, several potentially functional polymorphisms have been found. *These include the* rs13181 SNP, which corresponds to a Lys to Gln substitution at codon 751 that is associated with suboptimal DNA repair capacity [6]. The ERCC2 rs238406 variant, on the other hand, is a silent polymorphism (Arg156Arg) that might have an effect on the ERCC2 protein level through aberrant mRNA splicing rather than a direct enzymatic function [17].

XRCC1 is known to play a critical role in DNA single-strand break repair and in the base excision repair pathway. Defects in these pathways may result in accumulation of DNA damage, carcinogenesis, and may reduce chemotherapeutic sensitivity [18]. The XRCC1 Arg to Gln substitution at codon 399 (rs25487) in particular seems to affect oxaliplatin sensitivity by causing a functional change in the XRCC1 protein leading to impaired DNA repair activity [19].

Some studies have shown that *ERCC1, ERCC2,* and *XRCC1* polymorphisms may influence the clinical outcome in CRC patients treated with adjuvant [20] or palliative oxaliplatin-based chemotherapy [21, 22]. For instance, a recent meta-analysis showed that the *ERCC1-*rs11615 polymorphism is closely linked with the clinical outcomes of CRC patients treated with oxaliplatin-based chemotherapy [7]. However, published reports from individual studies are not always consistent, which in part may be due to small sample sizes.

This study aims to evaluate a possible link between SNPs in ERCC1 (rs11615), ERCC2 (rs238406 and rs13181), and XRCC1(rs25487) and the risk of CRC development, comparing 596 patients to 300 controls. The study further aims to assess the link between the SNPs and toxicity during treatment with 5-FU-based chemotherapy. Moreover, the same set of SNPs were analyzed in association with relapse-free survival (RFS) and OS of CRC patients.

## Methods

### Patients and controls

In total, 596 unselected, consecutive CRC patients treated at Sahlgrenska University Hospital/Östra between 1990 and 2006 were included. EDTA venous blood samples were collected from patients and 300 healthy blood donors. There was no gender difference between patients and controls (*p* = 0.9). Patients demographic and clinicopathological data was prospectively recorded based on medical records and follow up was done on a yearly basis. The tumour–node–metastasis staging system was used to classify tumours [23]. The regional ethical review board in Gothenburg approved the study and informed consent was obtained from all patients and controls.

### Toxicity

Patients were assessed for adverse events before each treatment cycle using the National Cancer Institute’s Common Terminology Criteria for Adverse Events (NCI-CTC AE) version 5.0 (https://ctep.cancer.gov/protocolDevelopment/electronic_applications/ctc.htm#ctc_50). Toxicities known to be related with the given treatment (diarrhoea, nausea, vomiting, stomatitis, fatigue, eye and skin reactions, leukopenia, thrombocytopenia, neutropenia, and neurotoxicity) were evaluated. A final toxicity evaluation was made at the end of treatment (complete or terminated due to toxicity) and the highest toxicity grade during treatment was noted. Toxicity was evaluated as a dichotomized variable: ‘no toxicity vs any toxicity’.

### Genotyping

Genomic DNA was extracted from EDTA venous blood samples using a magnetic bead extraction procedure on a Hamilton ML Star robot and quantified on agarose gel. The XRCC1 (rs25487), ERCC1 (rs11615), ERCC2 (rs13181), and ERCC2 (rs238406) genotypes were generated using TaqMan technology implemented on an ABI PRISM® 7900HT sequence detection system (Applied Biosystems, Foster City, CA). The assay numbers and context sequences were the following: XRCC1-rs25477, C__622564_10, GGGTTGGCGTGTGAGGCCTTACCTC**[C/T]**GGGAGGGCAGCCGCCGACGCATGCG; ERCC1-rs11615, C__2532959_20, TTACGTCGCCAAATTCCCAGGGCAC**[A/G]**TTGCGCACGAACTTCAGTACGGGAT; ERCC2-rs13181, C__3145033_10, TGCTGAGCAATCTGCTCTATCCTCT**[G/T]**CAGCGTCTCCTCTGATTCTAGCTGC; ERCC2-rs238406, C_8714009_10, CCTGCCCTCCAGTAACCTCATAGAA**[G/T]**CGGCAGTGGGGCAGGCTGGTGTCAT. Genotyping PCR reactions contained 2.5 μL of ABI TaqMan PCR Master Mix, 0.10 μL of ABI SNP assay-by-design master mix containing 900 nmol/L forward primer, 900 nmol/L reverse primer, 200 nmol/L VIC-labelled MGB probe, and 200 nmol/L FAM-labelled MGB probe, 10–20 ng of template DNA, and H_2_O to a final volume of 5 μL. Assay and TaqMan PCR mastermix was pipetted in a 384-well plate using a liquid-handling Biomek FX robot (Beckman Coulter Inc., San Diego, CA, USA). ABI PRISM® 7900HT Sequence Detection System (version 2.1) was used for TaqMan and fluorescent discrimination genotyping analyses. Unblinded control samples were included on each sample plate and were correctly genotyped by the SDS on 100% of occasions. Laboratory staff members employed in genotyping were blinded to clinical outcome.

### Statistical analysis

To test for Hardy-Weinberg equilibrium of the SNPs, a *Chi2* test was applied. Also, the association of SNPs with the risk of CRC development and toxicity during adjuvant or first line chemotherapy was analysed using *Chi2* test. The 5-year OS was calculated for all CRC patients (*n* = 596) from the date of surgery to date of all cause death, whereas the 5-year RFS was calculated for stage III CRC patients (*n* = 170) from the start of adjuvant treatment to date of CRC relapse or all cause death. Patients lost to follow-up were censored. The Kaplan–Meier method and log-rank test were used to evaluate the effects of polymorphisms and other covariates on survival analysis, reporting odds ratio (OR) and 95% confidence interval (CI). A *p-*value < 0.05 was considered to be significant. Analysis and plots were conducted using Stata MP v.14.

## Results

### Patients characteristics

Demographic and clinicopathological data are shown in Table 1. Mean age at diagnosis was 69.4 years. Most of the CRC patients were male (56.7%), had a colon cancer (57.6%) stage II or III (73.4%), and a well/moderately differentiated tumour (75.0%). The lymph node ratio (LNR), i.e., the number of positive lymph nodes divided by the number of analysed lymph nodes, was calculated. In 69.3% of patients at least 12 lymph nodes were examined and 52.1% of patients were found to be node positive. In rectal cancer patients, 21.7% received radiotherapy prior to surgery. Radical surgery was achieved in 86.3% and chemotherapy was administered in 39.6% of the patients. Relapse status was known for all but one of the stage I-III patients and relapse was confirmed in 32.1%. Three (0.5%) patients had unknown status at the last follow-up and 362 (60.7%) were deceased of which 225 (37.8%) were cancer-specific deaths.

**Table 1:** Patient characteristics (*n = 596*)

	*n (%)*
Age, mean (± SD) yrs	69.4 (± 12.1)
Gender (Male)	338 (56.7)
Tumor location	
Rectum	251 (42.4)
Left-sided colon	132 (22.3)
Right-sided colon	209 (35.3)
Tumor differentiation	
Well	22 (3.7)
Moderate	423 (71.3)
Poor	138 (23.3)
Mucinous	6 (1.0)
Stage	
I	62 (10.4)
II	203 (34.1)
III	234 (39.3)
IV	94 (15.8)
Lymph node count ≥12	413 (69.3)
Positive lymph node	293 (52.1)
Received preoperative radiotherapy^a^	130 (21.7)
Radically operated	509 (86.3)
Received chemotherapy^b^	236 (39.6)
Adjuvant therapy	171 (55.1)
First-line therapy	101 (25.4)
Confirmed response to therapy	74 (78.7)
Relapsed	160 (32.1)
Status at last follow-up	
Dead	362 (60.7)
Alive	231 (38.8)

NOTE- Unknown data: Tumor location for 4 patients; Tumor differentiation for 7 patients; Tumor stage for 3 patients; Radical operation for 6 patients; relapse for 1 patient, status at last follow-up for 3 patients. ^a^Only for rectal cancer patients; ^b^ 41 patients had both adjuvant and first-line therapy, response to therapy was measured only for 94 patients who had undergone first-line therapy

### Polymorphism distribution and its correlation with CRC risk

All the studied polymorphisms were in Hardy-Weinberg equilibrium in both CRC patients and healthy controls (Table 2). Table 3 summarizes the SNPs distribution in CRC cases and normal controls. As shown, the CC genotype of ERCC2-rs238406 and C allele of ERCC2-rs13181 were associated with a significantly increased risk of CRC, with odds ratios (95% CI) of 1.5 (1.1–2.0) and 1.4 (1.0–1.9), respectively (Table 3). To measure the combined effect of the two ERCC2 SNPs, patients and controls were grouped by having at least one favourable genotype (ERCC2-rs238406 C/A + A/A and ERCC2-rs13181 A/A) or unfavourable genotypes only (ERCC2-rs238406 C/C and ERCC2-rs13181 A/C + C/C), and compared. The odds ratio (95% CI) was increased to 1.8 (1.3–2.6) for patients with the unfavourable genotypes (Table 3). There were no statistically significant correlations between genotype distributions and age, gender, tumour location, tumour stage, tumour differentiation, lymph node metastasis, and other cancer characteristics assessed in the current study (data not shown).

**Table 2:** Genotype distributions in colorectal cancer patients and healthy controls

Gene	Group	Rs number	Locus/effect	Major homozygote	Heterozygote	Minor homozygote	Total *n*	Hardy-Weinberg *p*
XRCC1	Patients	rs25487	Arg399Gln	253 (G/G)	269 (G/A)	68 (A/A)	590	0.96
	Controls			128 (G/G)	136 (G/A)	36 (A/A)	300	1.0
ERCC1	Patients	rs11615	Asn118Asn	237 (T/T)	265 (C/T)	78 (C/C)	580	0.96
	Controls			122 (T/T)	151 (C/T)	27 (C/C)	300	0.12
ERCC2	Patients	rs238406	Arg156Arg	178 (C/C)	282 (C/A)	112 (A/A)	572	1.0
	Controls			70 (C/C)	162 (C/A)	65 (A/A)	297	0.29
ERCC2	Patients	rs13181	Lys751Gln	219 (A/A)	286 (A/C)	76 (C/C)	581	0.51
	Controls			138 (A/A)	125 (A/C)	31 (C/C)	294	0.94

NOTE- Missing data: XRCC1-rs25487 for 6 patients; ERCC1-rs11615 for 16 patients; ERCC2-rs238406 for 3 controls and 24 patients; ERCC2-rs13181 for 15 patients and 6 controls

**Table 3:** Comparison of polymorphisms between colorectal cancer patients and controls

	Total, *n (%)*	Patients, *n (%)*	Controls, *n (%)*	Odds ratio *(95% confidence interval)*	*p*
XRCC1-rs25487					
G/G	381 (42.8)	253 (42.9)	128 (42.7)		
G/A + A/A	509 (57.2)	337 (57.1)	172 (57.3)	1.0 (0.7–1.3)	0.95
ERCC1-rs11615					
T/T	359 (40.8)	237 (40.9)	122 (40.7)		
C/T + C/C	521 (59.2)	343 (59.1)	178 (59.3)	1.0 (0.7–1.3)	0.95
ERCC2-rs238406					
C/C	248 (28.5)	178 (31.1)	70 (23.6)		
C/A + A/A	621 (71.5)	394 (68.9)	227 (76.4)	1.5 (1.1–2.0)	0.01
ERCC2-rs13181					
A/C + C/C	518 (59.2)	362 (62.3)	156 (53.1)		
A/A	357 (40.8)	219 (37.7)	138 (46.9)	1.4 (1.0–1.9)	0.009
ERCC2-rs238406 AND ERCC2-rs13181					
Unfavourable: [ERCC2-rs238406 C/C] AND [ERCC2-rs13181 A/C + C/C]	227 (25.3)	172 (28.9)	55 (18.2)		
Favourable: [ERCC2-rs238406 C/A + A/A] AND/OR [ERCC2-rs13181 A/A]	670 (74.7)	424 (71.1)	246 (81.7)	1.8 (1.3–2.6)	0.001

### Polymorphisms and toxicity

Polymorphisms displaying a significant correlation with toxicity are presented in Table 4. In patients receiving adjuvant chemotherapy, the ERCC1-rs11615 genotype T/T was significantly associated with stomatitis (*p* = 0.03), and significantly more patients with the ERCC2-rs13181 C allele needed dose reduction compared to patients with the A/A genotype (*p* = 0.02). Among patients receiving first-line chemotherapy, significantly more patients with the ERCC1-rs11615 C allele suffered from nausea compared to those with the T/T genotype (*p* = 0.04). Also, eye reactions and thrombocytopenia were more common in patients with the ERCC2-rs13181 C allele compared to the A/A genotype (*p* = 0.006 and *p* = 0.004, respectively). Furthermore, the ERCC2-rs238406 C/C genotype was associated with a higher frequency of thrombocytopenia (*p* = 0.03).

**Table 4:** Polymorphisms and toxicity due to treatment of colorectal cancer patients

Polymorphism	Toxicity, *n* (%)	*p*
ERCC1-rs11615	Stomatitis^a^	
T/T	14/70 (20.0)	
C/T + C/C	8/94 (8.5)	0.03
ERCC1-rs11615	Nausea	
T/T	24/37 (64.9)	
C/T + C/C	48/58 (82.8)	0.04
ERCC2-rs13181	Eye reactions	
A/A	7/37 (18.9)	
A/C + C/C	27/58 (46.6)	0.**006**
ERCC2-rs13181	Thrombocytopenia	
A/A	8/36 (22.2)	
A/C + C/C	31/59 (52.5)	0.004
ERCC2-rs238406	Thrombocytopenia	
C/C	16/28 (57.1)	
C/A + A/A	23/67 (34.3)	0.03
ERCC2-rs13181	Dose reduction/discontinuation^a^	
A/A	38/70 (54.3)	
A/C + C/C	69/96 (71.9)	0.02

NOTE-^a^Patients who received adjuvant chemotherapy

### Survival analysis

Patients who died within 30 days after surgery (*n* = 3) were excluded from survival analysis. By the median follow-up time of 2313 days, 362/593 (61.0%) of the patients were deceased and 329/593 lived at least 5 years after the date of first surgery. Thus, the 5-year overall survival rate was 55.5% (95% CI, 51.4–59.5) and the 5-year cancer-specific survival was 67.5% (95% CI, 63.5–71.2). One hundred and seventy-one patients (28.7%) received adjuvant treatment (additional Table 1). Out of these patients, 156 were included in the 5-year RFS analysis (fifteen stage II patients were excluded from the RFS analysis, as was one stage IV patient down staged after surgery). Ninety-four of the 156 stage III CRC patients were relapse free at least 5 years after the start date of adjuvant treatment, resulting in a 5-year RFS of 60.3% (95% CI, 52.1–68.0).

**Fig. 1:**
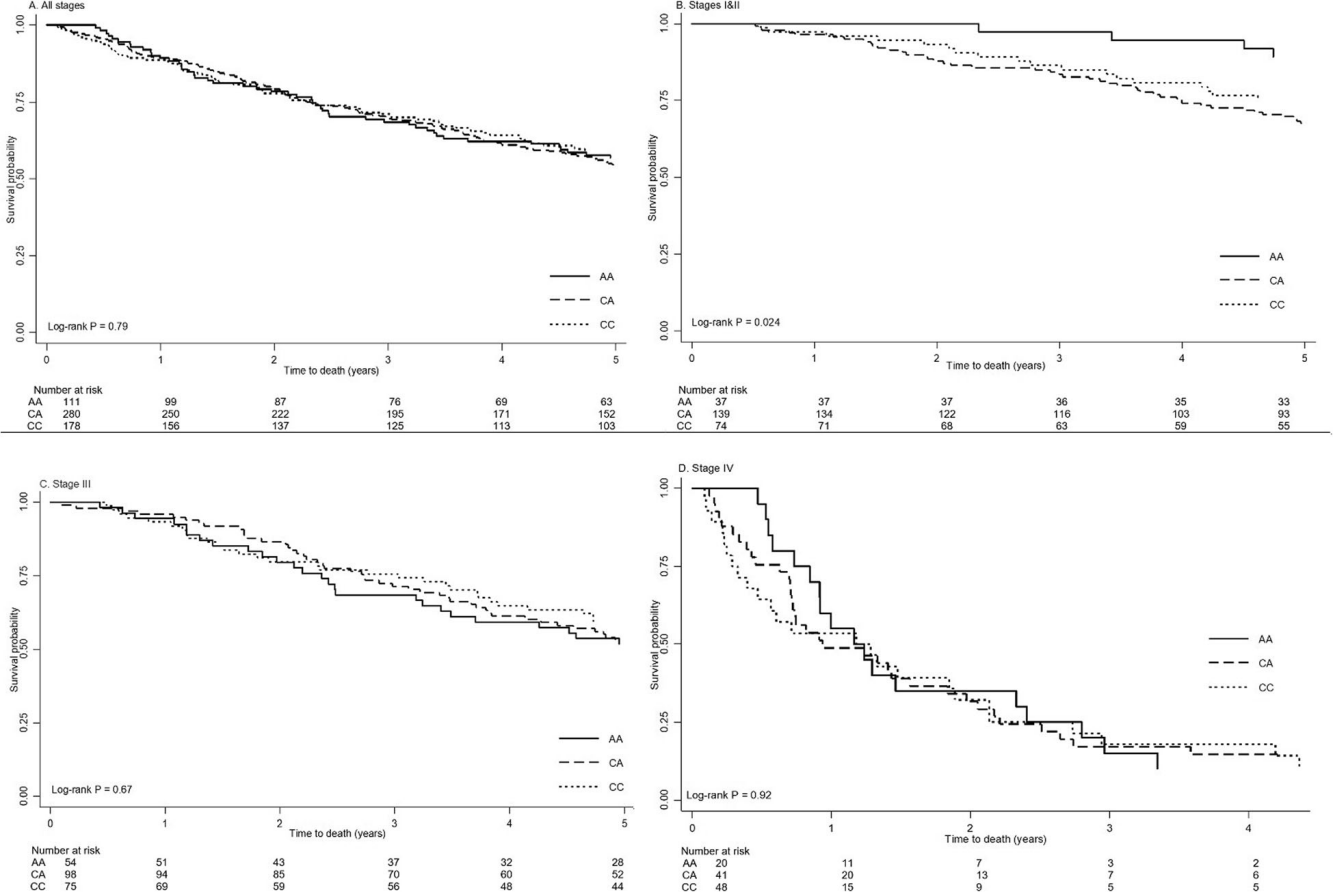
5-year overall survival of colorectal cancer by stage, ERCC2-rs238406 genotype

There were no significant associations of the studied SNPs with the 5-year OS of all stage CRC patients (Fig. 1a), nor with stage III or IV patients (Fig. 1c, d). However, the ERCC2-rs238406 polymorphism might affect survival in stage I & II CRC patients. As shown in Fig. 1b, significantly shorter 5-year OS was found to be associated with the ERCC2-rs238406 C allele (*p* = 0.02). There was no significant association with 5-year RFS of stage III patients (data not shown).

Univariate analysis evaluating prognostic factors affecting the 5-year overall survival, are shown in Table 5. There was a statistical significant difference in 5-year OS for patient age at diagnosis (*p* < 0.0001), cancer stage (*p* < 0.001), differentiation grade (*p* = 0.001), number of lymph nodes examined after resection (*p* < 0.001), node-positivity rate (*p* < 0.001), radical operation (*p* < 0.001), and year of surgery (*p* = 0.001). However, gender, primary tumour location, and the XRCC1-rs25487, ERCC1-rs11615, ERCC2-rs238406, and ERCC2-rs13181 polymorphisms were not statistically associated with 5-year OS (*p*’s > 0.05). Analyses of the combined effect of the four SNPs revealed no significant associations with OS.

**Table 5:** Prognostic factors of 5-year overall survival of colorectal cancer in univariate analysis

	Total *(n = 593)*	Events (*n = 268*)	Odds ratio *(95% confidence interval)*	*p*
Age, mean (±SD) yrs	69.4 (± 12.2)	72.4 (± 12.3)	1.03 (1.0–1.1)	< 0.0001
Gender, *n (%)*				
Male	337 (56.8)	157 (58.6)		
Female	256 (43.2)	111 (41.4)	0.9 (0.6–1.2)	0.4
Tumor location, *n (%)*				
Rectum	249 (42.3)	116 (43.8)		
Left-sided colon	132 (22.4)	53 (20.0)	0.8 (0.5–1.2)	0.2
Right-sided colon	208 (35.3)	96 (36.2)	1.0 (0.7–1.4)	0.9
Tumor differentiation, *n (%)*				
Well & moderate	445 (76.3)	183 (69.8)		
Poor	138 (23.7)	79 (30.2)	1.9 (1.3–2.9)	0.001
Stage, *n (%)*				
I & II	264 (44.8)	78 (29.1)		
III	232 (39.3)	105 (39.2)	1.9 (1.4–2.8)	0.0002
IV	94 (15.9)	85 (31.7)	22.5 (10.8–47.3)	< 0.0001
Lymph node positive, *n (%)*				
No	268 (45.2)	83 (31.0)		
Yes	325 (54.8)	185 (69.0)	2.9 (2.1–4.1)	< 0.0001
Lymph node count, *n (%)*				
< 12	183 (30.9)	99 (36.9)		
≥ 12	410 (69.1)	169 (63.1)	0.6 (0.4–0.8)	0.001
Radical operation, *n (%)*				
No	81 (13.8)	77 (29.3)		
Yes	506 (86.2)	186 (70.7)	0.03 (0.01–0.1)	< 0.001
Year of surgery, *n (%)*				
1990–2000	140 (23.6)	80 (29.8)		
2001–2006	453 (76.4)	188 (70.2)	0.5 (0.4–0.8)	0.001
XRCC1-rs25487, *n (%)*				
G/G	250 (42.6)	113 (43.1)		
G/A	269 (45.8)	117 (44.7)		
A/A	68 (11.6)	32 (12.2)	1.0 (0.7–1.4)	0.9
ERCC1-rs11615, *n (%)*				
T/T	236 (40.9)	105 (40.9)		
C/T	263 (45.6)	111 (43.1)		
C/C	78 (13.5)	41 (16.0)	1.1 (0.7–1.4)	0.9
ERCC2-rs238406, *n (%)*				
C/C	176 (30.9)	73 (29.0)		
C/A	281 (49.4)	130 (51.6)		
A/A	112 (19.7)	49 (19.4)	1.1 (0.6–1.2)	0.7
ERCC2-rs13181, *n (%)*				
A/A	219 (37.9)	99 (39.0)		
A/C	283 (49.0)	124 (48.8)		
C/C	76 (13.1)	31 (12.2)	1.1 (0.8–1.5)	0.7

## Discussion

In this relatively large case-control study of 596 CRC patients and 300 controls, we assessed the influence of genetic polymorphisms on CRC risk, treatment toxicity, and survival in CRC patients. The patient cohort was well-monitored with a follow-up period of at least 5 years.

### Polymorphisms and cancer risk

Data regarding the association between the investigated polymorphisms and CRC risk are controversial, which to a large extent is related to variability among populations. For each gene polymorphism, the minor allele varies greatly among ethnic groups. As an example, the XRCC1-rs25487 A allele ranges from 0.11 in the African population to 0.37 in European population [24], hence possibly contributing to different levels of susceptibility to CRC across populations. While previous studies on XRCC1-rs25487 confirmed the association of increased risk for CRC in particular among East Asians and Arab ethnicity [25–28], two meta-analysis studies, consistent with our results, suggested no association of this SNP and risk of CRC [29, 30]. Further large studies in well characterized cohorts are therefore needed to establish an association between the XRCC1-rs25487 polymorphism and CRC risk and how it varies in different populations.

The frequency of the ERCC1-rs11516 T > C polymorphism also varies greatly among different ethnical populations. The reference T allele, which seems to be associated with a higher mRNA expression compared to the C allele [16], has a frequency of 0.62 and 0.26 in European and East Asian populations, respectively [6, 24]. Thus, the T allele is the major allele in European populations but the minor allele in Asian populations. This may explain some of the discrepant results regarding this SNP. For instance, some Chinese [26, 31] and Norwegian [32] studies assessed the ERCC1-rs11615 polymorphism and CRC risk but found no significant correlations [33], in accordance with the findings of the present study. In contrast, another Asian study showed that the ERCC1-rs11615 genotype T/T contributed to a marginally increased CRC risk compared to CC genotype [34].

Our findings indicate that the ERCC2-rs238406 CC genotype and/or the C allele of ERCC2-rs13181 confer a significantly increased risk of CRC. The OR obtained was even stronger when combining ERCC2-rs238406 and ERCC2-rs13181. Our results support findings in a Spanish population suggesting that the rs13181 heterozygote is linked to higher risk of CRC compared to AA or CC genotypes [35]. In contrast, the risk of CRC was significantly increased with the ERCC2-rs13181 A allele in one Romanian study [36]. Other reports suggest that the CC genotype is associated with decreased CRC risk in American [37] and Iranian [38] populations. Also, many studies, including one meta-analysis, which assessed the relation between the ERCC2-rs13181 polymorphism and CRC risk in multiple populations failed to find any link [39, 40]. Thus, more knowledge on the mechanisms of the ERCC2 variants is needed to clarify the implications of the present data.

### Polymorphisms and toxicity

Treatment-induced toxicity sometimes results in dose reduction or termination of treatment [41]. In our study of patients receiving adjuvant FLV or FLOX therapy, the ERCC1-rs11615 genotype T/T was significantly associated with stomatitis, and among patients receiving first-line chemotherapy, the ERCC1-rs11615 C allele was associated with nausea. However, we could not find any association between ERCC1-rs11615 and haematological toxicity, as was shown in a Chinese population with non-small lung cancer for the ERCC1-rs11615 genotype T/T to be correlated with severe leukopenia [42]. Since the T allele is associated with a higher protein expression compared to the C allele [16], presumably resulting in a higher repair capacity, these results are contradictory and need to be addressed in a larger cohort of patients.

In our study, significantly more patients with the ERCC2-rs13181 C allele had eye reactions and thrombocytopenia and needed dose reduction more often compared to patients with the A/A genotype. We also found that the ERCC2-rs238406 C/C genotype was associated with a higher frequency of thrombocytopenia. Haematological toxicity has also been reported in a previous study, where the ERCC2-rs13181 C allele was significantly associated with an increased risk of FOLFOX-induced toxicity [43]. These results can partly be explained by the fact that both the ERCC2-rs13181 C allele and the ERCC2-rs238406 C/C genotype are associated with reduced enzyme activity and suboptimal DNA repair, leading to increased sensitivity of normal cells to DNA-damaging agents like oxaliplatin [6, 17, 41]. No association between XRCC1 polymorphism and any of the investigated toxicity parameters was however found. Although these results are interesting, they need to be confirmed in other large patient cohorts. It would be also of value to analyse the combined effect of the studied SNPs on toxicity in a larger group.

Discrepancies in the association between polymorphisms and toxicity among studies might in addition to being dependent on ethnicity, be due to gender differences as reported in a recent publication by Ruzzo et al. [44]. However, the impact of gender or type of chemotherapy given could not be assessed in our study due to insufficient number of patients in each toxicity subgroup. Even larger homogenous cohorts in terms of treatment regimens and gender distribution are needed to provide reliable data for subgroup analysis.

### Polymorphisms and survival

In general, neither 5-year RFS nor OS were associated with any of the polymorphisms in the present study with the exception of the ERCC2-rs238406 C allele that was associated with significantly shorter 5-year OS among stage I and II CRC patients. These results are in agreement with one study in a Nordic population showing that patients with the ERCC2-rs238406 A/A genotype had a significantly longer progression-free survival compared to patients with the C/A and C/C genotypes [41]. There was no significant difference, however, in the OS. Indeed, this polymorphism may reduce ERCC2 protein levels by altering mRNA stability [42] and a reduced ERCC2 protein activity in patients with the ERCC2-rs238406 A/A genotype may lead to an increased sensitivity to DNA-damaging drugs like oxaliplatin and therefore a better progression-free survival [41].

Although we did not find any association between the ERCC2-rs13181 and OS, this SNP has been suggested to be a prognostic predictor for CRC [45] and one American study showed that CRC patients carrying the ERCC2-rs13181 C/C genotype displayed poor survival [46]. Also, a meta-analysis indicated that the ERCC2-rs13181 C allele was linked with poorer OS in Caucasians [6].

In contrast to our findings, the ERCC1-rs11615 T allele has been associated with reduced response to treatment and shorter OS in oxaliplatin-treated Asian CRC patients [6], probably due to high expression of ERCC1, and may be a predictive factor for CRC [47]. Nevertheless, the European Society for Medical Oncology guidelines are currently against the use of ERCC1 expression status in therapeutic decisions on oxaliplatin use in routine clinical practice due to inconsistent results [48].

Our results are consistent with the literature in failing to identify a significant prognostic effect of the XRCC1 SNP in metastatic CRC patients. Most studies found no strong association of XRCC1 genotype with clinical outcome [49–52]. Nonetheless, other studies have shown that CRC patients who carried at least one A allele were at an increased risk of developing resistance to oxaliplatin-based treatment [19, 53]. Likewise, the prognostic effect of the XRCC1–25487 polymorphism has been confirmed with shorter disease-free survival in patients with A/A genotype [21].

Although the present study is relatively large, the number of patients did not permit robust analysis in selective sub-groups. For instance, it would be interesting to study the impact of gene variants in stage III and stage IV patients grouped by different treatment regimens. However, we used a well-defined patient cohort with a long follow-up time which provided potentially clinically reliable information.

## Conclusions

Both SNPs in ERCC2 were associated with a significantly increased risk of CRC. In addition, the ERCC2- rs238406 was linked to OS in early stage CRC and both ERCC2-rs238406 and ERCC2-rs13181 were associated with toxicity during first line treatment. Specifically, the ERCC2-rs238406 CC genotype was associated with thrombocytopenia whereas the ERCC2-rs13181 C variant was correlated with thrombocytopenia as well as eye reactions. The ERCC1-rs11615 genotype T/T was significantly associated with stomatitis during adjuvant treatment, while among patients receiving first-line chemotherapy, the ERCC1-rs11615 C allele was associated with nausea. The results add support to previous findings that SNPs in ERCC1 and ERCC2 have a prognostic and predictive value in clinical management of CRC.

## Supplementary information

**Additional file 1: Table 1.** shows patient distribution according to treatment regimens, e.g., FLV: 5-FU & leucovorin; FLIRI: FLV & irinotecan; FLOX: FLV & oxaliplatin in first-line (*n* = 171) and adjuvant (*n* = 101) chemotherapy groups.

## Data Availability

The datasets used and/or analyzed during the current study are available from the corresponding author on reasonable request.

## Abbreviations

CRC: Colorectal cancer
OS: Overall survival
5-FU: 5-fluorouracil
FLV therapy: 5-FU plus leucovorin
SNP: Single nucleotide polymorphism
DNA: Deoxyribonucleic acid
ERCC1: Excision repair cross-complementing group 1
ERCC2: Excision repair cross-complementing group 2
XRCC1: X-ray repair cross-complementing group 1
RFS: Relapse-free survival
EDTA: Ethylenediaminetetraacetic acid
NCI-CTC AE: National Cancer Institute’s Common Terminology Criteria for Adverse Events
PCR: Polymerase chain reaction
OR: Odds ratio
CI: Confidence interval
LNR: Lymph node ratio
FLOX: 5-fluorouracil plus leucovorin plus oxaliplatin
FLIRI: 5-fluorouracil plus leucovorin plus irinotecan
