# Supplementary material for: Association of DNA repair gene variants with colorectal cancer: risk, toxicity, and survival
Source: BMC Cancer. 2020 May 12;20:409. doi: 10.1186/s12885-020-06924-z (PMC7216326; doi:10.1186/s12885-020-06924-z)
Supplement: Supplementary file 1 — Additional file 1: Table 1. shows patient distribution according to treatment regimens, e.g., FLV: 5-FU & leucovorin; FLIRI: FLV & irinotecan; FLOX: FLV & oxaliplatin in first-line (n = 171) and adjuvant (n = 101) chemotherapy groups. [file 12885_2020_6924_MOESM1_ESM.docx]

| **Additional Table 1** Patient distribution according to treatment regimens | | |
| --- | --- | --- |
| Treatment type | First-line, *n (%)* | Adjuvant, *n (%)* |
| FLV | 35 (34.7) | 149 (7.1) |
| FLIRI | 34 (33.7) | 1 (0.6) |
| FLOX | 21 (20.8) | 21 (12.3) |
| FLOX/FLIRI + Avastin/Cetuximab | 11 (10.9) | 0 (0.0) |
| Total | 101 | 171 |
| NOTE- FLV: 5-FU & leucovorin; FLIRI: FLV & irinotecan; FLOX: FLV & oxaliplatin | | |
